# Does the Urinary Proteome Reflect ccRCC Stage and Grade Progression?

**DOI:** 10.3390/diagnostics11122369

**Published:** 2021-12-16

**Authors:** Lucia Santorelli, Martina Stella, Clizia Chinello, Giulia Capitoli, Isabella Piga, Andrew Smith, Angelica Grasso, Marco Grasso, Giorgio Bovo, Fulvio Magni

**Affiliations:** 1 Clinical Proteomics and Metabolomics Unit, School of Medicine and Surgery, University of Milano—Bicocca, 20854 Vedano al Lambro, Italy; l.santorelli@campus.unimib.it (L.S.); Martina.Stella@bruker.com (M.S.); clizia.chinello@gmail.com (C.C.); isabella.piga@unimib.it (I.P.); andrew.smith@unimib.it (A.S.); 2 Centre of Biostatistics for Clinical Epidemiology, School of Medicine and Surgery, University of Milano—Bicocca, 20854 Vedano al Lambro, Italy; giulia.capitoli@unimib.it; 3 Urology Unit, S. Gerardo Hospital, 20900 Monza, Italy; angelica_grasso@yahoo.it (A.G.); m.grasso@asst-monza.it (M.G.); 4 Pathology Unit, Vimercate Hospital, 20871 Vimercate, Italy; giorgio.bovo@asst-vimercate.it

**Keywords:** clear cell renal cell carcinoma, proteomics, urine, tumor grade, tumor stage

## Abstract

Due its ability to provide a global snapshot of kidney physiology, urine has emerged as a highly promising, non-invasive source in the search for new molecular indicators of disease diagnosis, prognosis, and surveillance. In particular, proteomics represents an ideal strategy for the identification of urinary protein markers; thus, a urinomic approach could also represent a powerful tool in the investigation of the most common kidney cancer, which is clear cell Renal Cell Carcinoma (ccRCC). Currently, these tumors are classified after surgical removal using the TNM and nuclear grading systems and prognosis is usually predicted based upon staging. However, the aggressiveness and clinical outcomes of ccRCC remain heterogeneous within each stratified group, highlighting the need for novel molecular indicators that can predict the progression of these tumors. In our study, we explored the association between the urinary proteome and the ccRCC staging and grading classification. The urine proteome of 44 ccRCC patients with lesions of varying severity was analyzed via label-free proteomics. MS data revealed several proteins with altered abundance according to clinicopathological stratification. Specifically, we determined a panel of dysregulated proteins strictly related to stage and grade, suggesting the potential utility of MS-based urinomics as a complementary tool in the staging process of ccRCC.

## 1. Introduction

Renal Cell Carcinoma (RCC) comprises a heterogeneous group of tumors of which the most frequent (70–80%) and aggressive morphotype is clear cell RCC (ccRCC) [1,2]. In contrast to other urological tumors, ccRCC is both chemo-resistant and radio-resistant and tends to metastasize before presenting any local sign or symptom; in fact, in more than one third of patients, the tumor is already metastatic at diagnosis. As such, surgical resection seems to be the only effective therapy for this disease to date [3].

ccRCC is a malignancy of particular interest for clinical investigations because of its associated inter-tumor and intra-tumor heterogeneity [4], high recurrence and progression rates, and lack of effective non-invasive diagnostic and prognostic indicators [5].

Routinely, ccRCC lesions are classified by considering two principal clinical features according to the recommendation of the World Health Organisation (WHO) classification [5]. One is represented by the stage feature, assigned following the TNM classification system, that combines anatomical factors such as (T) tumor size and its location (venous invasion, renal capsular invasion, or adrenal involvement), (N) lymph node, and (M) distant metastasis involvement [6]. The other is the grade feature, assessed using the nuclear grading system, which is based primarily on the cytological and morphological description of nucleoli by microscope observation. This system was proposed at the 2013 International Society of Urological Pathology (ISUP) conference and accepted by the WHO in 2016 [7], replacing the previously used Fuhrman grading system [8]. To date, tumor staging and histological grading classifications are recognized as the main parameters for ccRCC prognosis and are widely used in clinical practice.

Although this system is well established and represents the key tool in the armory of pathologists, the need for new molecular markers that are able to assist in ccRCC diagnosis and prognosis remains essential. In particular, this is due to both grading and staging stratifications employing descriptive parameters that rely on the subjective evaluation of the physician [9]. Consequently, this approach is not always capable of univocally classifying the lesions, especially in those cases that are borderline between two successive classes of tumor groups. Accordingly, the discovery of specific molecular markers of ccRCC could constitute a feasible means of reducing uncertainty surrounding its diagnosis and provide additional information that is able to guide treatment decisions.

Additionally, regarding the molecular mechanisms underpinning the progression of ccRCC, they are still unclear and studies that can provide a more in-depth understanding of the biological process involved in this advancement will be beneficial for its management.

In this context, proteomics strategies have been widely used and biofluids have represented the sample type of choice due to them being easily and noninvasively obtainable [10]. The new concept of a liquid biopsy has been extensively investigated as a valid alternative to the classical solid biopsies in biomarker discovery [11,12,13,14,15]. In the last decades, different proteomics and metabolomics approaches based on mass spectrometry (MS) have been used to investigate urinary content for the purpose of biomarker discovery and, in particular, studying kidney diseases with a special focus on tumor development, progression, and staging [16,17,18,19,20].

However, excluding some exceptions [21,22], most of the studies employing biofluids focused on the differences between patients with ccRCC and healthy controls [23]. Therefore, in this study, we investigated the proteome of ccRCC patients with lesions of varying severity in order to obtain a deeper insight into molecular alterations associated with the progression of this cancer. The results obtained by a label free-MS based method resulted in the detection of a set of urinary proteins associated with different ccRCC lesions and potentially with the aggressiveness of the disease.

## 2. Materials and Methods

### 2.1. Patient Selection

Forty-four patients from San Gerardo hospital (Monza, Italy) were recruited between 2011 and 2016, and all subjects had signed informed consent. The local ethical committee (Comitato Etico Azienda Ospedaliera San Gerardo, Monza, Italy, BPCR 24-02-2011) approved all protocols and procedures. Analysis was performed in agreement with the Declaration of Helsinki. The diagnosis was performed after careful histopathological evaluation, including tumor size and position (pT), grading (ISUP classification system), sarcomatoid features, vascular invasion, tumor necrosis, presence of distant metastasis, and involvement of lymph nodes (stage).

### 2.2. Sample Collection and Preparation

The second urine sample of the morning, before total or partial nephrectomy, was collected into sterile urine tubes. The samples were then centrifuged at 1000× *g* for 10 min at 4 °C and stored at −80 °C until the day of the analysis. An amount of 6 mL of the stored supernatant was concentrated by centrifugation on a 30 kDa cut-off filter (Amicon Ultra-4 mL 3 kDa, Millipore, Bedford, MA, USA) for 10 min at 4 °C.

Protein concentration was determined with the BCA method (Microplate BCA ™ protein Assay Kit, Thermo Scientific, Sunnyvale, CA, USA) and 200 μg of protein from each of the urine samples was used for tryptic digestion [24,25]. Briefly, proteins were first reduced by incubation with 50 mM DL-Dithiothreitol (Sigma Aldrich, Buchs, Switzerland) and then alkylated for 30 min with Iodoacetamide 100 mM (Sigma Aldrich, Buchs Switzerland). Overnight digestion was performed on 30 kDa filters (Amicon Ultra-500 μg 30 kDa, Millipore) following the filter-aided sample preparation (FASP) protocol, adding trypsin from porcine pancreas (Proteomics Grade, BioReagent, Dimethylated) in a ratio of 1:100 with respect to the initial protein concentration. The digested peptides were eluted by three steps of centrifugations at 14,000× *g* (10 min) and acidified. The resulting peptide solution was desalted and concentrated using μ-C18 ZiptipTM pipette tips (Millipore Corp, Bedford, MA, USA) following the standard protocol provided by Millipore. Purified samples were resuspended in 98% H_2_O: 2% acetonatrile: 0.1% trifluoroacetic acid and analyzed by nUHPLC-MS/MS.

### 2.3. nUHPLC-MS/MS Analysis

LC-ESI-MSMS analysis was performed using a Dionex UltiMate3000 rapid separation (RS) LC nano system (Thermo Scientific, Sunnyvale, CA, USA) coupled with an UHR-nESI-qTOF (Impact HDTM, Bruker Daltonics Germany equipped with a Captivespray nanoBooster). Approximately 1.4 μg of protein was loaded onto μ-precolumn (Dionex, Acclaim PepMac100 C18, cartridge, 300 μm i.d. × 5 mm, 5 μm) and then separated by an analytical C18 column (Dionex, 75 μm ID, Acclaim PepMac100, C18, 2 μm). A multistep gradient with phase A (0.1% formic acid) and phase B (0.1% formic acid/80% acetonitrile) from 4% to 66% was run for 240 min. Eluted peptides were analyzed in Data-Dependent Acquisition mode, and MS/MS data were acquired by targeting precursors (*m*/*z* 300–2000 range) with a charge state between +2 and +5 and with at least 1575 counts (fixed cycle time of 5 s) for fragmentation, obtained by collision-induced dissociation. MS scans as well and MS/MS data were recorded in centroid. Raw MS and MS/MS data were corrected using both an internal calibration (lock mass *m*/*z* 1221.9906) and a calibration segment, based on a 10 mM sodium formiate cluster solution (15 min before each run), prior to being converted and deconvoluted to XML file format using DataAnalysis software (Bruker Daltonics, Bremen, Germany).

### 2.4. Data Processing

Protein identification was obtained with PEAKS Studio 8.5 (Bioinformatics Solutions Inc., Waterloo, ON, Canada [26]). Trypsin was set as the enzyme used for the digestion, with 1 as a maximum acceptable number of missed cleavages, carbamidomethylation as fixed modification, and 20 ppm and 0.05 Da as mass tolerance for MS and MS/MS tolerance, respectively. Swiss-prot (accessed July 2017; 555′100 sequences; 198′754′198 residues) was used as database. The maximum false discovery rate (FDR) for peptide spectral match (PSM) was set to 1%, retention time window of 2 min was established for features matching between runs, and a minimum of one sequence-unique peptides was required for identification.

Protein abundances were calculated using the three most abundant unique peptides, normalized against the total ion current and only those proteins detected with at least two unique peptides and in at least 50% of the samples were used for protein quantification. Files containing information regarding protein abundance in the form of statistical matrices (samples as rows and proteins as columns) were exported from PEAKS Studio 8.5 and imported into an in-house developed software. For each protein, statistical evaluation was performed as follows in order to retrieve a list of differentially expressed proteins. First, patients were grouped according to the levels of a chosen outcome attribute (e.g., the grade), then proteins were considered to have a different abundance (ratio > 1.5 and <1.5) when the *p*-value yielded by the Mann–Whitney test was less than 0.05. Due to the high degree of biological variability, no further statistical correction was performed. Functional analysis was carried out for the proteins of interest: KEGG pathway term clustering using ClueGO v2.5, Clupedia v1.5, and Cytoscape network analysis framework was performed [27].

## 3. Results

Conventional histopathological evaluation of tumors, such as stage and grade of the lesions, has prognostic significance. However, the molecular mechanisms involved in tumor development and progression are not well understood. Therefore, the possibility to obtain information regarding molecular changes associated with tumor lesions has been investigated using easily accessible urine samples.

### 3.1. Clinical Data and Study Design

Urine samples were collected from 44 patients (26 males, 18 females; median age at diagnosis of 66 and 69 years, respectively, and mean tumor mass of 5.54 ± 3.33 cm) with a proven diagnosis of ccRCC (Table 1 and Table S1). All diagnoses were determined by three specialists (G.B., anatomopathologist; M.G. and A.G., urologists). Patients were classified according to both the 2010 (7th edition) and 2017 (8th edition) AJCC TNM staging and 2016 grading systems [6,7]. We grouped patients according to the 8th AJCC edition for the statistical analysis. Specific focus was given to both grade (Low Grade (LG) = G1 and G2; High Grade (HG) = G3 and G4) and stage (stage 1 and stage > 1) values in order to evaluate proteomic signatures of different lesions, using a liquid biopsy investigation-based approach.

### 3.2. Qualitative Evaluation and Network Analysis: Relevant Pathways of ccRCC

**Figure 1:**
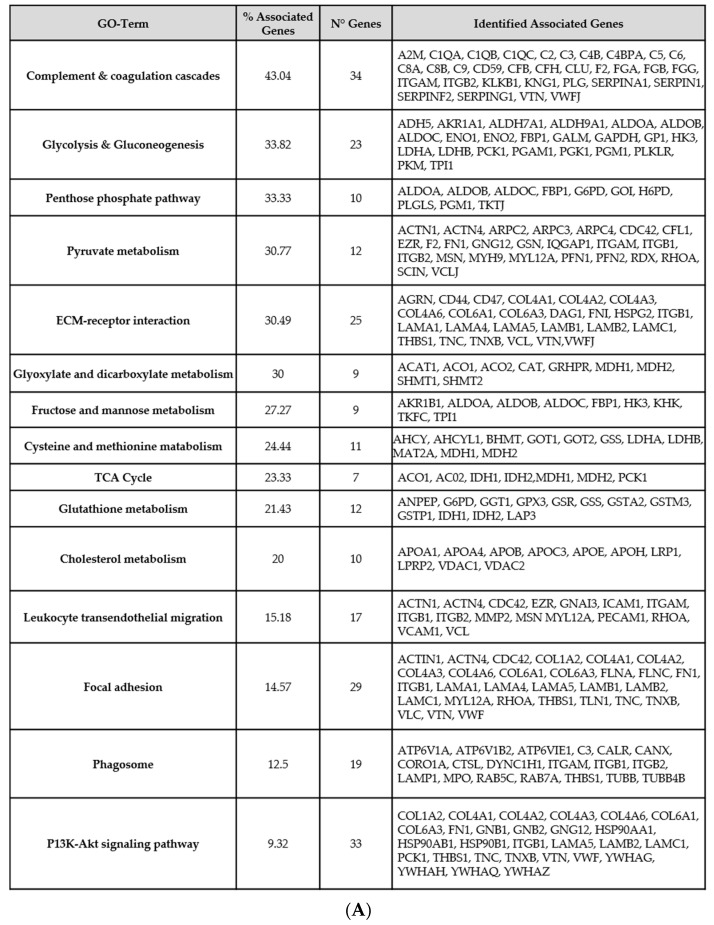

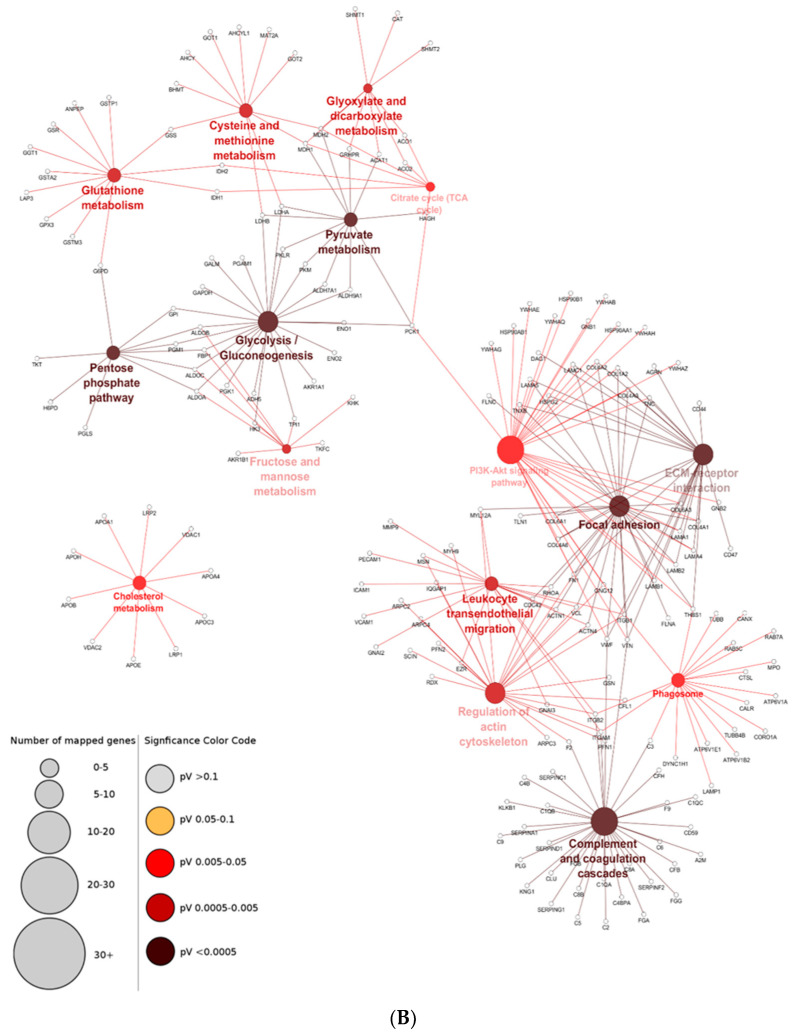
Top Pathways involved in ccRCC progression. (**A**) Annotations of proteins and pathways shared by urine and tissue of ccRCC patients. (**B**) Network showing the main pathways and their significance (light to dark red, *p*-value < 0.05).

Following the FASP protocol, samples were analyzed by nUHPLC-MS/MS and a total of 21,640 peptides sequences, corresponding to 1609 proteins, were identified with an FDR on PSM of 1% and at least one unique peptide (Table S2). The proteins identified in urine were compared with those identified on tissue in a previous study [28], and more than 500 proteins were observed to be present in both urine and tissues specimens. Moreover, pathways represented by common proteins were investigated using the KEGG database. The processes that were associated with the presence of ccRCC lesions are presented in Figure 1A,B.

Primarily, GO term enrichment analysis highlighted glycolysis/gluconeogenesis, the pentose phosphate pathway, and pyruvate metabolism as the chiefly represented processes with a total of 52 identified proteins found to be associated with them. A number of urinary proteins coded by hub genes involved in the cellular reactions of carbohydrates (fructose and mannose metabolism, nine proteins), lipids (cholesterol metabolism, 10 proteins), and amino acids (cysteine and methionine metabolism, 11 proteins) were also detected. Additionally, functional annotation analysis indicated the presence of proteins involved in immune response and coagulation cascade in urine.

### 3.3. Quantitative Evaluation: Urinary Proteins Varying According to Different Grade and Stage

We further investigated the correlation between urinary protein levels and tumor features (stage and grade), focusing on urinary proteome alterations that possibly reflected morphological changes of the lesions.

Initially, the influence of the grade on the urinary proteome was evaluated. Patients with different tumor grades were divided into two groups, HG (G3-G4) and LG (G1-G2) tumors, and, between them, 39 proteins were identified as differentially expressed. Among them, 10 were down and 29 upregulated in HG compared to the LG group (Table 2).

Moreover, in order to highlight alterations of the urinary proteome related to different stages, we also evaluated the differences between stage 1 and stage > 1. As result, 79 proteins with a statistically significant variation in their abundances were detected: 16 proteins were downregulated while 63 were upregulated in stage > 1 versus stage 1 patients (Table 3).

**Figure 2:**
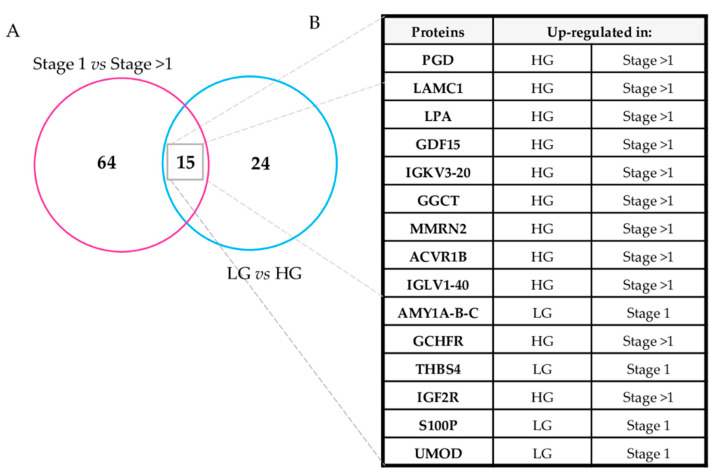
Proteins altered in the urine of ccRCC patients according with stage and grade lesions. (**A**) Venn diagram of the urinary proteins altered according to the stage (Stage 1 vs. Stage > 1) and grade (LG versus LG). (**B**) List of 15 proteins commonly altered in grade and stage comparisons.

Urinary proteins altered according to the stage (stage 1 versus stage > 1), and grade (LG versus HG) were also compared (Figure 2A) and the 15 proteins commonly altered showed the same quantitative pattern: Four were overexpressed both in LG and in stage 1 samples while, conversely, 11 were overexpressed in HG and stage > 1 lesions (Figure 2B). This observation suggest that the overlapping altered protein profiles were associated with the aggressiveness of disease when stratified by both stage and grade.

In order to better understand which of the urinary protein alterations are specifically correlated with these clinical hallmarks of disease (grade and stage), we proceeded to sub-classify our cohort of patients by considering each feature individually.

**Figure 3:**
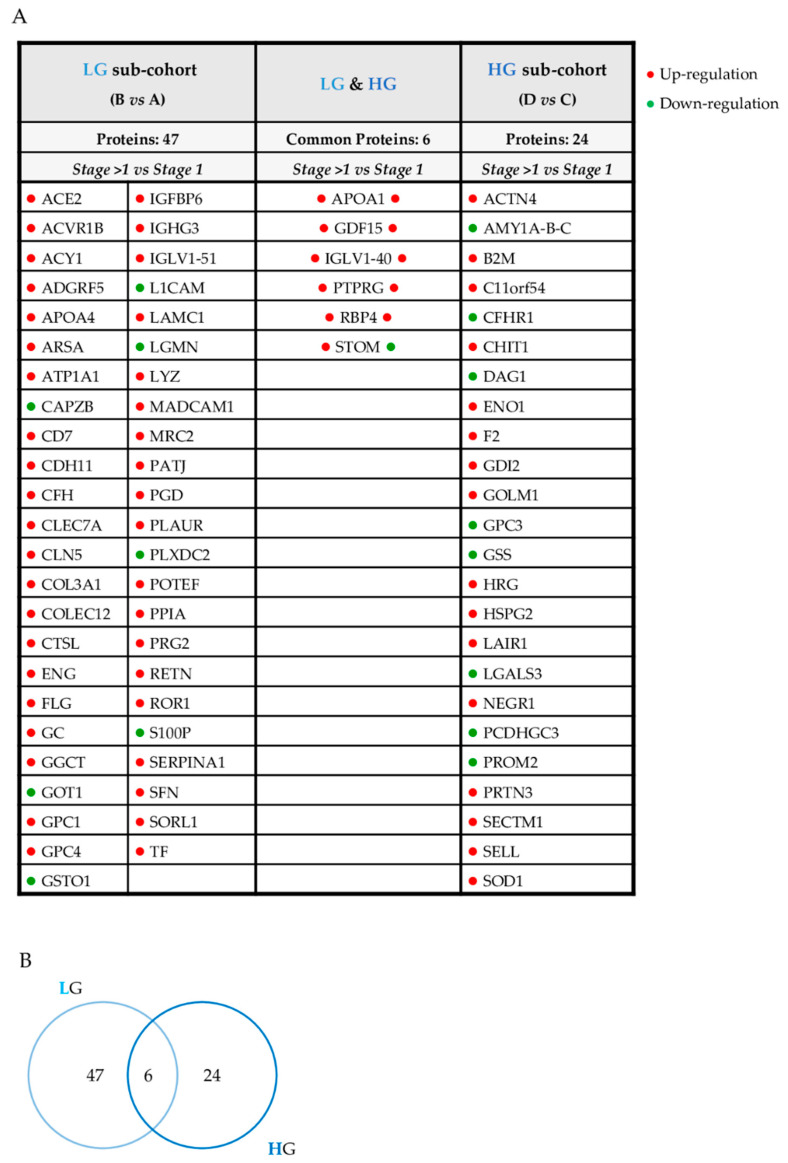
Urinary proteins varied according to stage. List (**A**) and Venn diagram (**B**) of urinary proteins altered according to HG and LG, considering only stage as a variable feature.

Initially, we primarily focused on the influence of the stage stratification on the urinary proteome. We compared patients with LG and stage 1 lesions with those with LG and stage > 1 lesions (groups A and B); then, compared patients with stage 1 and stage > 1 lesions at HG (groups C and D). We determined six proteins that were upregulated in advanced stage with respect to stage 1 when grade classification was excluded as a confounding factor (Figure 3A,B).

**Figure 4:**
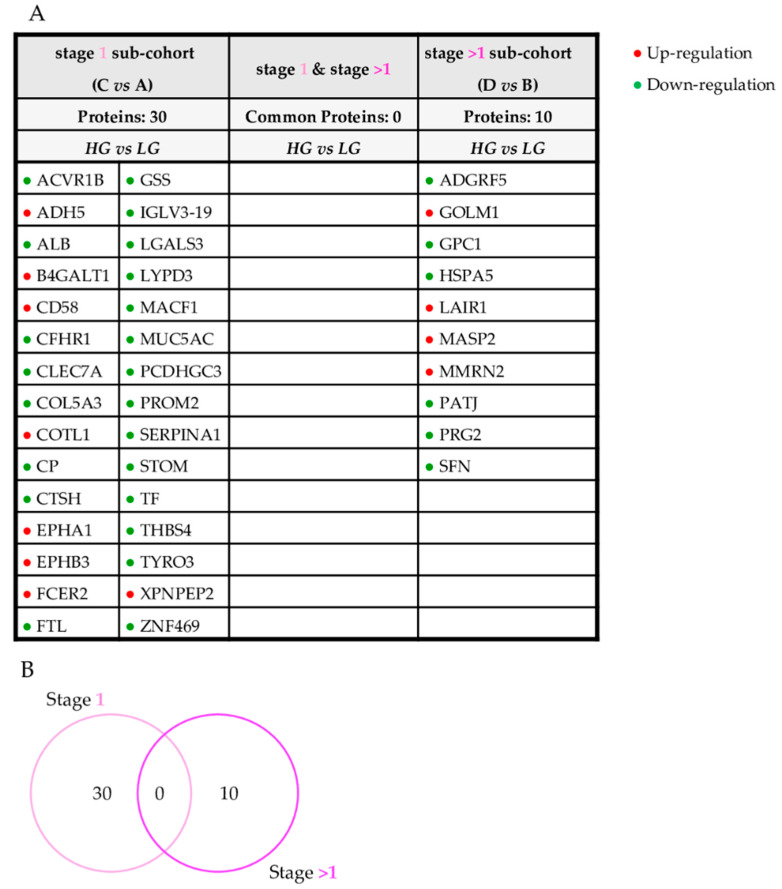
Urinary proteins varied according to grade. List (**A**) and Venn diagram (**B**) of urinary proteins altered according to stage 1 and stage > 1, considering only grade as a variable feature.

Subsequently, we investigated how the expression of urinary proteins reflected tumor grade stratification. Firstly, we compared patients with lesions at different grades: LG (group A) versus HG (group C) at stage 1 and LG (group B) versus HG (group D) at stage > 1 (Figure 4A,B). This comparison did not highlight any proteins that correlated specifically with the grade independently from the stage.

## 4. Discussion

### 4.1. Urine as a Molecular Portrait of ccRCC

Proteomics studies of ccRCC using different MS technologies have been applied to a variety sample types, including tumor cell lines and different types of human samples, such as serum, tissue, and urine [29,30,31,32]. Using a combined MS approach (MALDI MSI and nLC-ESI-MS/MS), we have previously described the proteomic profile of different ccRCC tumor grades using histology-guided regions of tissue. By using this strategy, we highlighted several protein signals with an intensity trend associated with different tumor grades [28]. We also explored the field of post-translation modifications, detecting alterations in the N-glycosylation pattern of proteins in urine of ccRCC patients with different stages [33].

In light of these previous findings, we hypothesized that the urinary proteome may reflect both the morphological features (stage and grade) used by the pathologist to define the progression of the tumor. For this purpose, we analyzed the urine of ccRCC patients, stratified according to grade (LG and HG) and stage (stage 1 and stage > 1). Firstly, we identified more than 1600 urinary proteins, of which 500 were previously detected in ccRCC tissues, supporting the hypothesis that urine also has the potential to provide key information regarding the functionality and pathological state of the kidney.

GO network analysis of these common proteins highlighted the involvement of various pathways, including glucose oxidation and the pentose phosphate pathway (Figure 1A,B). It is reported that these metabolic pathways are the preferential mechanisms adopted by the tumor cells to degrade glucose, even in the presence of adequate oxygen availability from mitochondrial oxidation. This type of energy production shift is noted as Warburg’s effect, and it is considered the principal hallmark of RCC carcinogenesis [34]. Indeed, ccRCC is widely accepted as a metabolic disease in which the typically mutated genes have dysregulation effects on metabolic pathways involved in oxygen or nutrient sensing [35]. Our findings were strictly consistent with the latest research performed in the field.

Moreover, information provided by urine and tissue had a considerable overlap: proteins detected in both ccRCC urine and tissue samples are also involved in the activation of immune response in line with the data present in the literature. For instance, Tian et al. identified components of complement activation pathway, such as C1QA, C1QB, and C1QC, as key enriched genes [36]. The same trend was observed in our GO analysis, providing further encouraging evidence that urine can serve as an adequate tool for investigating ccRCC progression.

### 4.2. ccRCC Urinary Proteome Mirrors Grade and Stage Classification

In order to enlighten the possible correlation between urine proteome and histopathological tumor classification, we performed a quantitative label-free analysis. Firstly, the urine samples were grouped according to grade stratification. Thirty-nine proteins with a statistically significant differential abundance between the HG and LG classes were observed (Table 2). Secondly, quantitative analysis was repeated on the same samples, grouping them according to stage, highlighting 79 dysregulated proteins between stage 1 and stage > 1 (Table 3). When combining the two lists, 15 proteins emerged as commonly varied in stage and grade (Figure 2A,B), suggesting that their abundance may equally reflect both histopathological features.

However, evaluating both the stage and grade simultaneously could act as a confounding factor. In order to examine this aspect in greater detail, the entire cohort of patients was reclassified, considering a single morphological feature at a time. Therefore, four additional comparisons were performed: patients with the same grade were compared to each other by varying only the stage feature, and patients with the same stage were compared to each other considering as variable only the grade feature (Figure 3 and Figure 4, Table S3).

It was noteworthy that nine of the fifteen proteins, initially found to be commonly altered between stage and grade, reappeared in these further comparisons. As such, this panel of proteins could represent a good indicator of ccRCC progression. For instance, GDF15 and IGLV1-40 were upregulated when stage features (early and advanced) were compared, even when adjusted for different grades. Accordingly, we can speculate that their expression primarily reflected the stage, but the influence of the grade still remains relevant. In fact, these proteins are statistically significant even in the general comparison HG vs. LG (Table S3). The same consideration is relevant for proteins AMY A-B-C, GGCT, LAMC1, MMRN2, and S100P. They emerged as significant in three comparisons: one that involved the grade and two that involved the stage. AMY A-B-C and S100P are consistently downregulated while LAMC1, GGCT, and MMRN2 are consistently upregulated. Furthermore, the abundance levels of this group of proteins seemed to reflect primarily the stage.

To the best of our knowledge, there are no studies investigating protein changes in urine associated with ccRCC lesions at different stages and grades simultaneously. However, some of the proteins that we found to be quantitatively varied in the urine of ccRCC affected patients have been reported as altered in diverse cancers types (Table 4).

Among them, a remarkable interest is represented by laminin γ1 chain (LAMC1), a core structural protein present in the basement membrane of several organs, including the kidney. Immunohistochemical analysis of kidney specimens from patients with Chronic Kidney Disease (CKD) indicated an increased presence of LAMC1 in the glomerular basement membrane that could reflect an acceleration of its remodelling. A fragment of LAMC1 has also been detected in the in serum and urine of CKD patients with a high risk of progression [37]. Furthermore, it is also reported that the LAMC1 gene is upregulated in ccRCC tissue with respect to what is normal [38]. All these data suggest that LACM1 plays a crucial role in tumor activity and cancer progression, consistent with our finding. Indeed, in the urine from our cohort, we detected LAMC1 to be upregulated in stage > 1 (fc 2.14, *p*-value 0.003) and HG groups (fc 1.62, *p*-value 0.017). Moreover, when patients were reclassified, fixing one histopathological feature at a time, LAMC1 was once again upregulated in stage > 1 and in LG (fc 2.77, *p*-value 0.03) (Table 4).

Another important player in cancer cell proliferation has been demonstrated to be the glutamylcyclotransferase enzyme (GGCT). Involved in glutathione metabolism, it was found to be overexpressed in a variety of cancers, including uterine cervix, lung, and colon [39]. Our data are in good agreement with the study of Li et al. where they showed the overexpression of GGCT in high-grade serous ovarian cancer [40]. Even in our case, we detected an upregulation of GGCT in patients stratified as stage > 1 (fc 1.90, *p*-value 0.005) and HG (fc 1.54, *p*-value 0.03). The same pattern of overexpression was confirmed for the stage > 1 when we considered only the LG sub-cohort (fc 1.88, *p*-value 0.04) (Table 4).

Finally, Growth Differentiation Factor 15 (GDF15), a stress-inducible cytokine, also has important implications in the context of ccRCC. It plays key roles in prenatal development, inflammation, regulation of cellular responses to stress signals, and tissue repair [41,42]. GDF-15 has been reported as a biomarker in several kinds of pathologies, including kidney disease [43]. High circulating GDF-15 levels showed a significant correlation with a faster decline of renal function in patients with diabetes mellitus type 1, IgA nephropathy, and CKD stages 1–4 [44,45,46], suggesting its possible relevance as a marker of progression in ccRCC. Indeed, Traeger et al. already investigated this possibility, demonstrating that high GDF-15 levels in the plasma of RCC patients were associated with metastasis, relapse, and poor survival [47]. In our investigation, high GDF-15 levels in the urine of ccRCC patients were associated with advanced cancerous state, stage > 1 (fc 3.84, *p*-value 0.0002) and an HG (fc 2.64, *p*-value 0.035). In addition, the protein was once again upregulated in stage > 1 patients, even when the cohort was divided into HG (fc 9.22, *p*-value 0.03) and LG groups (fc 1.87, *p*-value 0.03) (Table 4).

Once a panel of urinary proteins for which its variation could be linked to the ccRCC stage was determined, each clinical parameter was separately considered. In performing this, many of the dysregulated proteins were present only in initial comparisons (Figure 2, Table 2 and Table 3).

However, the additional statistical analysis (Figure 2 and Figure 3, Table S3), stage > 1 vs. stage 1 in LG; stage > 1 vs. stage 1 in HG; HG vs. LG in stage 1; and HG vs. LG in stage > 1 highlighted a further set of altered proteins. Six of them were varied in patients with tumors of different stage (Figure 3, Table S3). We considered these proteins as molecular urinary markers capable of primarily reflecting the stage since they were varied in the comparison stage > 1 vs. stage 1 for both HG and LG sub-groups. Conversely, the comparison of HG vs. LG, fixing sub-populations stage 1 and stage > 1, did not show any protein alterations that could be primarily associated with the grade of the lesion (Figure 4, Table S3).

Notwithstanding, a subset of proteins, such as ADGRF5, PATJ, PRG2, and SFN, also showed a complementary expression trend and suggested a more complex molecular scenario. They were upregulated in LG of the stage > 1 subpopulation and in the stage > 1 of the LG subpopulation (Table 4). We speculated that they could specifically reflect the combination of early grade and advanced stage staging.

Our results are in accordance with those already reported in the literature. For instance, Pals1-Associated Tight Junction protein (PATJ) is a highly expressed protein in kidney epithelia and is involved in the maintenance of cell polarity [48]. By using a whole exome sequencing study, Wang et al. detected several newly somatic mutations of the PATJ gene in renal tissue of RCC patients. They also revealed a strong correlation between the mutated form of the PATJ gene and the positive expression of Programmed Death Ligand 1 (PD-L1), already known as a biomarker of response to the immunotherapy in many cancers, including RCC [49]. It is also interesting to mention that stratifin (SNF), a highly conserved ubiquitously expressed protein, is associated with many different cellular processes and directly linked to cancer onset and progression. [50,51]. In our analysis, both PATJ and SNF were upregulated in the urine of ccRCC patients stratified as advanced stage and early grade (Table 4 and Table S3). Their presence could represent an additional piece in the molecular puzzle of tumorigenic and metastatic processes in ccRCC.

The hypothesis that the urine proteome reflects all the theoretical combinations of stage and grade could also explain the characteristic quantitative pattern of GOLM1 and LAIR1. Both were upregulated in stage > 1 when considering only the HG sub-cohort and up-regulated in HG, when considering only the stage > 1 sub-cohort (Table 4 and Table S3). Golgi Membrane protein 1 (GOLM1) is a transmembrane glycoprotein of Golgi cisternae, commonly expressed in epithelial cells of normal tissues [52], and several studies have pinpointed its implication in carcinogenesis. For instance, in prostate and breast cancers, GOLM1 works as an oncogene by inducing cancer cell growth, migration, and invasion, inhibiting cell apoptosis [53]. Leukocyte Associated Immunoglobulin-like Receptor 1 (LAIR1) is a transmembrane glycoprotein expressed in almost all cells of the immune system. It is reported that the overexpression of LAIR-1 is associated with advanced grades in oral squamous cell carcinoma [54]. More interestingly, LAIR1 was found to be significantly upregulated in RCC with respect to normal tissue and is likely to promote cell proliferation by upregulation of Akt phosphorylation in RCC [55].

Moreover, a further two proteins with possible implication are THBS4 and XPNPEP2. They were both determined to be downregulated in HG in the general comparison of HG vs. LG but upregulated in the HG group when the stage 1 feature was fixed (Table 4 and Table S3). In this case, the interdependence of the two histopathological features is evident. Depending on the group of patients considered, in fact, the two tumor features differently influence the expression trend of the proteins. Nevertheless, we could still assume that the presence of a complementary expression trend might reflect the advanced grade, especially in combination with stage 1 lesions.

Along these lines, it is interesting to report the altered abundance of Thrombospondin-4 protein (THBS4). It is an adhesive glycoprotein, part of the extracellular matrix, and it is involved in proliferation, attachment, adhesion, and cell migration as well as spreading in various types of malignancies [56]. For instance, Chen et al. demonstrated that THBS4 upregulation was positively linked with increased malignant potential and a poor clinical outcome in gastric cancer [57]. Overexpression of THBS4 was also highly correlated with advanced stage vascular invasion in hepatocellular carcinoma [58]. We observed THBS4 to be downregulated in ccRCC patients with LG lesions in the comparison when only considering grade (fc −1.61, *p*-value 0.02) but upregulated in the HG lesion group (fc 2.43, *p*-value 0.03) when the sub-cohort of stage 1 patients was evaluated. We, therefore, suggest that THBS4 plays a role in advanced ccRCC grade only when HG and early stage lesions are co-present (Table 2).

Finally, a similar consideration can be made for the proteins CLEC7A, CP, and CTSH. They were upregulated in the HG cohort of patients that were classified on the sole basis of lesion grade; however, when statistical analysis was limited to the stage 1 patient group, these proteins were in fact upregulated in the LG condition (Table S3). Among them, CLEC7A has particular biological significance. While also known as dectin-1, CLEC7A was originally identified as a pattern-recognition receptor expressed on dendritic cells [59]. In fact, it was demonstrated that its expression on dendritic cells and macrophages enhances the recognition of N-glycans on cancer cells, aiding natural killer cells in depleting tumor cells [60]. In this regard, Xia et al., using immunohistochemistry, showed that CLEC7A expression was strongly correlated with a higher stage of ccRCC. They also demonstrated that high CLEC7A expression was an independent predictor for disease-free recurrence as well as disease-free and overall survival [61]. Our analysis pinpointed that CLEC7A levels were statistically significant in the urine of ccRCC patients. Specifically, a consistent upregulation was observed in LG tumors when stage (1) is fixed (fc 3.38, *p*-value 0.016) and in stage > 1 when the LG lesion is fixed (fc 2.15, *p*-value 0.019) (Table 4 and Table S3). In accordance with the tissue proteome [60], urinary CLEC7A levels appeared to correlate with the higher ccRCC stage. Additionally, its presence in the urine of ccRCC patients also appeared to correlate with a lower grade. Moreover, deregulation emerged only when a specific sub-cohort (LG and stage 1) of patients was considered.

## 5. Conclusions

In this study, we highlight that the urinary proteome could represent a precious source of information regarding the molecular understanding of ccRCC progression. Collectively, we revealed a set of proteins with altered urinary expression according to tumor metastasis. Several of them were already known to be involved in carcinogenesis, tumor progression, and aggressiveness in RCC and in other cancer types. These findings suggest that such proteins profiles could be helpful in characterizing this malignancy in a non-invasive manner.

Indeed, the urinary proteome of ccRCC patients primarily mirrors stage classification and partially provides information regarding the grade of the tumor, operating as a liquid biopsy of kidney cancer progression. As such, it was possible to generate a panel of proteins for which its abundance was specifically linked to each of these tumor features. Taken together, these findings represent a valuable starting point when seeking to develop a complementary molecular tool to support TNM and nuclear grading systems, improving individual prognostic evaluations of ccRCC patients.

In the future, we plan to enlarge the patient cohort in order to assess the possible association with comorbidities and to collect urine from post-surgery subjects in order to enforce the robustness of our results.

## Figures and Tables

**Table 1:** Clinicopathological characteristics of the 44 patients enrolled in the study, grouped according to grade and stage classifications.

Group	Number of Patients	Gender (Male–Female)	Age at Diagnosis (Mean)	Greatest Tumor Dimension (cm)
Stage 1, pT1	19	11–7	68	3.5
Stage > 1, pT3	25	12–13	67	7.1
Low Grade (LG)	24	12–12	66	4.3
High Grade (HG)	20	14–6	69	6.9
Stage 1—Low Grade ^1^ (A)	15	7–8	65	3.4
Stage > 1—Low Grade (B)	9	5–4	67	5.8
Stage 1—High Grade ^2^ (C)	4	4–0	71	3.7
Stage > 1—High Grade (D)	16	10–6	67	7.8

^1^ Low grade (LG) = G1 and G2; ^2^ High grade (HG) = G3 and G4.

**Table 2:** Statistically significant variation of urinary proteins levels according to the grade.

HG vs LG			
Total Varied Proteins: 39			
Proteins: 29			Proteins: 10
● ACVR1B	● GGCT	● MMP9	● AMY1A-B-C
● ART3	● GNS	● MMRN2	● ANXA11
● AZGP1	● HP	● ORM1	● CAT
● CLEC7A	● HSPA1B	● ORM2	● HSP90AA1
● COL3A1	● IGF2R	● PGD	● PCDH12
● CP	● IGKV3-20	● PZP	● PLAU
● CRIM1	● IGLL1	● RNASET2	● S100P
● CTSH	● IGLV1-40	● SDC1	● THBS4
● GCHFR	● LAMC1	● TNFRSF14	● UMOD
● GDF15	● LPA		● XPNPEP2

● Up-regulation ● Down-regulation.

**Table 3:** Statistically significant variation of urinary proteins levels according to the stage.

STAGE >1 vs STAGE 1			
Total Varied Proteins: 79			
Proteins: 63			Proteins: 16
● ACY1	● GCHFR	● MUC5B	● AMY1A-B-C
● ACVR1B	● GC	● NEGR1	● AOC1
● ALDH1A1	● GDF15	● NGFR	● C6
● APP	● GGCT	● PATJ	● CORO1A
● AQP1	● GPC4	● PGD	● FGL2
● B2M	● HAVCR2	● PLAUR	● GSTO1
● CD300LF	● HRG	● PPIA	● IST1
● CD7	● HSPG2	● PRTN3	● KRT2
● CD93	● HYAL1	● PTPRG	● KRT84
● CDH11	● IGF2R	● RBP4	● PLD3
● CFB	● IGKC	● RETN	● PLXDC2
● CFHR2	● IGKV2-28	● ROR1	● PROM2
● CLU	● IGKV3-20	● SECTM1	● PTPRJ
● CNDP2	● IGKV3-15	● SELL	● S100P
● COL15A1	● IGKV4-1	● SERPINA1	● THBS4
● COLEC12	● IGLV1-40	● SFN	● UMOD
● COMP	● IGLV1-51	● SHISA5	
● CTSL	● LAMC1	● SOD1	
● ENG	● LPA	● SPINT2	
● F2	● LYZ	● TFF2	
● FBLN2	● MMRN2	● TMPRSS2	

● Up-regulation ● Down-regulation.

**Table 4:** Selection of dysregulated urinary proteins in ccRCC already reported as altered in other diseases.

Detected Proteins	Reflected Features in Our ccRCC Patients	Other Types of Pathologies	Specimen
GDF15	Primarily stage	Diabetes mellitus type 1	Plasma
		Membranous IgA Nephropathy	
		CKD	
		RCC	
GGCT	Primarily stage	Uterine cervix carcinoma	Tissue
		Lung carcinoma	
		Colon carcinoma	
		Ovarian carcinoma	
LAMC1	Primarily stage	CKD	Tissue
		ccRCC	Serum
			Urine
PATJ	Early grade	RCC	Tissue
	Advanced stage		
SNF	Early grade	Lung carcinoma	Tissue
	Advanced stage		
GOLM1	Advanced grade	Lung squamous cell carcinoma	Tissue
	Advanced stage		
LAIR1	Advanced grade	Oral squamous cell carcinoma	Tissue
	Advanced stage	RCC	
THBS4	Interdependence	Gastric cancer	Tissue
	grade and stage	Hepatocellular carcinoma	
CLEC7A	Interdependence	ccRCC	Tissue
	grade and stage		

## Supplementary Materials

The following are available online at https://www.mdpi.com/article/10.3390/diagnostics11122369/s1, Table S1: Stage and Grade grouping for each individual case; Table S2: List of the identified proteins in all the 44 urine specimens; Table S3: Abundance levels of dysregulated urinary proteins in ccRCC patients.
